# Microbiota potentialized larvicidal action of imidazolium salts against *Aedes aegypti* (Diptera: Culicidae)

**DOI:** 10.1038/s41598-019-52687-4

**Published:** 2019-11-07

**Authors:** Harry Luiz Pilz-Junior, Alessandra Bittencourt de Lemos, Kauana Nunes de Almeida, Gertrudes Corção, Henri Stephan Schrekker, Carlos Eugenio Silva, Onilda Santos da Silva

**Affiliations:** 1 Department of Microbiology, Immunology and Parasitology, Institute of Basic Health Sciences, Universidade Federal do Rio Grande do Sul, Rua Sarmento Leite 500, Porto Alegre, RS 90050-170 Brazil; 2 Laboratory of Technological Processes and Catalysis, Institute of Chemistry, Universidade Federal do Rio Grande do Sul, Av. Bento Gonçalves 9500, Porto Alegre, RS 91501-970 Brazil

**Keywords:** Bacteria, Microbiology

## Abstract

Mosquitoes are important vectors of pathogens due to their blood feeding behavior. *Aedes aegypti* (Diptera: Culicidae) transmits arboviruses, such as dengue, Zika, and Chikungunya. This species carries several bacteria that may be beneficial for its biological and physiological development. Therefore, studying the response of its microbiota to chemical products could result in vector control. Recently, imidazolium salts (IS) were identified as effective *Ae*. *aegypti* larvicides. Considering the importance of the mosquito microbiota, this study addressed the influence of IS on the bacteria of *Ae*. *aegypti* larvae. After exposition of larvae to different IS concentrations, the cultured microbiota was identified through culturomics and mass spectrometry, and the non-cultivated microbiota was characterized by molecular markers. In addition, the influence of the IS on axenic larvae was studied for comparison. There was an alteration in both cultivable species and in their diversity, including modifications in bacterial communities. The axenic larvae were less susceptible to the IS, which was increased after exposing these larvae to bacteria of laboratory breeding water. This highlights the importance of understanding the role of the larval microbiota of *Ae*. *aegypti* in the development of imidazolium salt-based larvicides. Such effect of IS towards microbiota of *Ae*. *aegypti* larvae, through their antimicrobial action, increases their larvicidal potential.

## Introduction

*Aedes aegypti* (Diptera: Culicidae) is an important mosquito species that is involved in the maintenance, replication and transmission of pathogenic agents, such as dengue, urban yellow fever, Zika and Chikungunya^1^. The incidence of these arboviruses has increased gradually through the decades, causing a major impact on health worldwide^2,3^. Currently, there is only an effective vaccine against the yellow fever virus. In the case of the registered dengue vaccine, it has an efficiency of only 60%^4^. Therefore, controlling and preventing dengue and other arboviruses still depends on vector handling. Within this perspective, the World Health Organization established normatives for controlling larvae and adult populations of *Ae*. *aegypti*, recommending the use of a combination of chemical and biological products^5^.

Current control methods have problems such as a high cost for the simultaneous biological and chemical control, and a low specificity for target organisms, being, therefore, toxic for non-target organisms, including the human being. Besides, several *Ae*. *aegypti* populations distributed worldwide showed resistance to the most used insecticides like the organophosphate Temephos, carbamates and pyrethroids^6^. For these reasons, the search for new products specific for target organisms with low impact in the environment is crucial for effective vector control.

**Figure 1:**
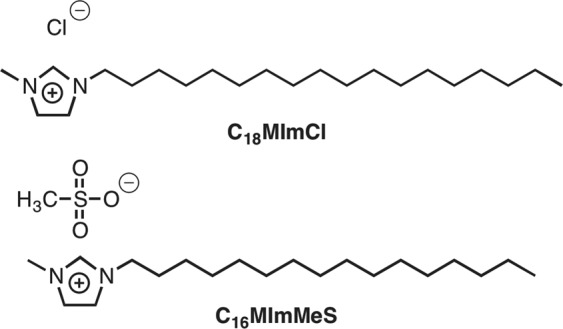
IS with previously identified larvicidal activity against *Ae*. *aegypti*.

Recently, Goellner *et al*. demonstrated the efficacy of two imidazolium salts (IS), 1-*n* octadecil-3-methylimidazolium chloride (**C**_**18**_**MImCl**) and 1-*n*-hexadecil-3-methylimidazolium methanesulfonate (**C**_**16**_**MImMeS**), in the control of *Ae*. *aegypti* larvae (Fig. 1)^7^. A broad variety of biological properties have been reported for these IS, including antibacterial activities^8–10^. This has been ascribed to Coulombic interactions between IS ions and phosphatidylethanolamine groups of the bacterial cell membrane, causing a disjunction of the membrane that leads to lysis and cell death^11^. As a consequence, the understanding of the role of *Ae*. *aegypti* microbiota is of importance when treating the larvae with IS. This mosquito species can develop ecological interactions with other species, mostly with microorganisms. The microbiota has performed a crucial role in the evolutionary process of organisms, influencing in several biological functions, such as nutrition, reproduction, development and immunity. In addition, some bacteria can mediate or stop the settlement of pathogens and, consequently, their transmission^12,13^. Herein, we describe for the first time the effect of the *Ae*. *aegypti* larvae microbiota on the larvicidal potential of the IS presented in Fig. 1.

## Results

### Identification of bacterial isolates in larvae treated with IS

Larvae were exposed to three previously defined concentrations (LC_99_, LC_50_ and LC_20_)^7^ of the two IS presented in Fig. 1. All IS treatments caused changes in the larvae microbiota. Besides, some species disappeared and others appeared, compared to the control group. The identified bacterial species are listed in Table 1.

**Table 1:** Bacterial diversity identified by culturomics in *Aedes aegypti* larvae treated with LC_99_, LC_50_ and LC_20_ IS solutions. x Means occurrence and — absence of the species after treatment.

*Microbiota* (*Control*)	C_18_MImCl			C_16_MImMeS		
	LC_99_	LC_50_	LC_20_	LC_99_	LC_50_	LC_20_
*Serratia marcescens*	—	—	x	x	—	x
*Enterobacter asburiae*	—	—	x	x	x	x
*Stenotrophomonas altophilia*	—	—	x	x	x	x
*Klebsiella oxytoca*	x	x	x	x	x	x
*Delftia acidovorans*	—	x	x	x	x	x
*Enterobacter kobei*	x	x	x	x	x	x
*Providencia rettgeri*	—	x	x	x	x	x
*Aeromonas* *sp*.	x	—	x	x	x	x
*Pseudomonas sp*.	x	x	x	—	x	x
*Burkholderia sp*.	—	—	—	—	—	x
*Microbacterium sp*.	x	x	—	—	—	—

A total of eleven distinct bacterial morphotypes were isolated and identified by using MALDI-TOF mass spectrometry, being seven and four through species and genus level, respectively. **C**_**18**_**MImCl** decreased the number of bacterial species with an increasing IS concentration. When larvae were treated with **C**_**16**_**MImMeS**, the number of bacterial species was relatively concentration independent, showing only a slightly lower number of species than the control group.

Ribosomal Intergenic Space Analysis (RISA) enabled building phylogenetic trees based on the position and the number of amplified DNA fragments, and the Shannon’s (H′) diversity analysis through the intensity of DNA fragments. Larvae treated with LC_99_ and LC_50_ IS concentrations were evaluated and compared to the control group. To analyze the bacterial communities in larvae exposed to IS, the genetic similarity between samples was calculated through the Dice coefficient and the data grouped through the unweighted pair group method with arithmetic mean (UPGMA).

**Figure 2:**
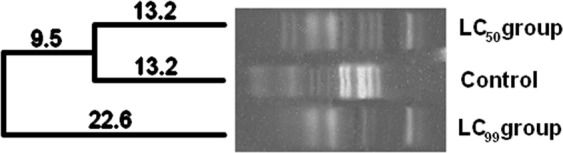
Phylogenetic tree generated through RISA analyses of the bacterial communities treated with **C**_**16**_**MImMeS** in different concentrations. Tree built using the Dice coefficient and grouping by the UPGMA algorithm. The distances represent dissimilarity. Samples derive from the same experiment. The full-length gel is presented in the Supplementary Information as Fig. S5.

**Figure 3:**
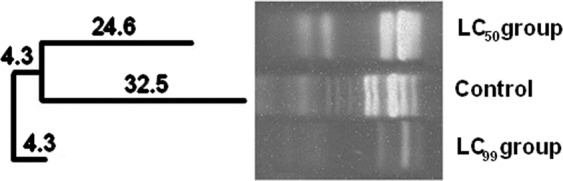
Phylogenetic tree generated through RISA analyses of bacterial communities treated with **C**_**18**_**MImCl** in different concentrations. Tree built using the Dice coefficient and grouping by the UPGMA algorithm. The distances represent dissimilarity. Samples derive from the same experiment. The full-length gel is presented in the Supplementary Information as Fig. S6.

The Figs 2 and 3 show the phylogenetic trees after the exposition to **C**_**16**_**MImMeS** and **C**_**18**_**MImCl**, respectively. Larvae treated with the LC_50_ of **C**_**16**_**MImMeS** and the control group showed the same coefficient of dissimilarity. Its LC_99_ affected the bacterial community, observing a greater distance and the creation of a new branch in the phylogenetic tree (Fig. 2). Almost the same happened to larvae treated with **C**_**18**_**MImCl**. Now, although the coefficients of dissimilarity were different between the groups, the bacterial population did not distance itself from the main branch for the LC_99_ treatment, even though a new one was created (Fig. 3).

**Figure 4:**
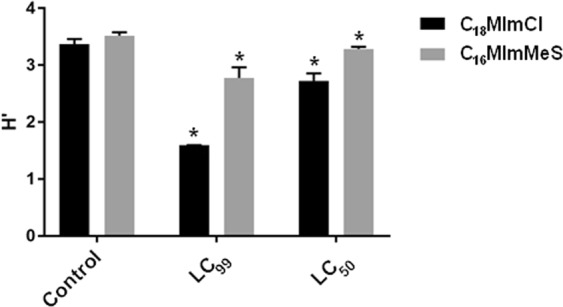
Shannon diversity index (H′) determined for the microbiota after the treatments of larvae with different IS concentrations. The bars represent standard deviations. *Represents significant difference compared to the control.

In both IS treatments, the Shannon’s (H′) diversity decreased as its concentration was increased (Fig. 4). **C**_**16**_**MImMeS** presented a less pronounced difference in diversity when compared to **C**_**18**_**MImCl**.

### Effect of IS in axenic larvae

**Figure 5:**
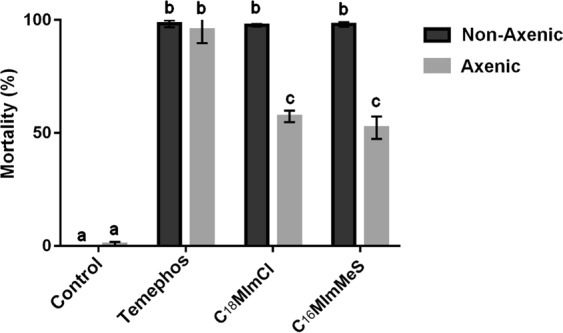
Mortality of larvae (axenic and non-axenic) after 48 h of exposure to IS (5 µg/mL) or Temephos (6 µg/L). The control is without IS and Temephos. Letters represent significant differences between treatments. The bars represent standard deviations.

The effect of the IS and Temephos was verified in axenic larvae. This axenic condition was proven by the absence of any bacterial growth. Figure 5 shows the mortalities of the exposed non-axenic and axenic larvae. Although Temephos caused the same high mortality of ≥80% in both tested groups, **C**_**18**_**MImCl** and **C**_**16**_**MImMeS** showed a significant (p < 0.05) reduction in mortality in axenic larvae.

### Effect of IS over re-colonized axenic larvae

**Figure 6:**
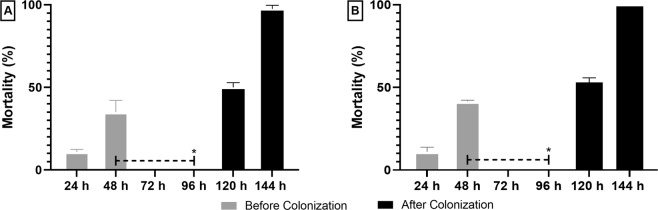
Mortality of *Aedes aegypti* larvae after being re-colonized and exposed to IS: (**A**) (**C**_**18**_**MImCl**); (**B**) (**C**_**16**_**MImMeS**). The dashed line (between 48 and 96 h) represents the time that axenic larvae that survived the first exposure to IS were re-colonized with microorganisms from standard laboratory breeding water. *(96 h) Represents the time that re-colonized larvae were re-exposed to IS. The bars represent standard deviations.

Exposure of axenic larvae to an IS LC_99_ resulted in the survival of half of the larvae after 48 h. Those axenic larvae, when exposed to the standard non-sterile laboratory breeding conditions, became colonized by bacteria. In addition, they became susceptible to the effect of IS. The axenia of the larvae was checked before and after the re-colonization. In the first case, no bacterial growth was observed and, after re-colonization, the turbidity in the medium after the maceration of larvae indicated the presence of bacteria. After 48 h of post-exposure and re-colonization, mortality rates higher than 95% were obtained for both treatments (Fig. 6).

## Discussion

The *Ae*. *aegypti* larvae treated with IS showed eleven species of bacteria, all of them belonging to the phylum Proteobacteria and being mostly gram-negative. Similar results have been described in the identification of cultivable normal microbiota of some culicide larvae^14–17^. A study conducted by Apte-Deshpande *et al*.^18^ demonstrated the presence of seven bacterial genera in the intestines of a laboratory reared *Ae*. *aegypti* larvae (*Aeromonas*, *Burkholderia*, *Enterobacter*, *Microbacterium*, *Pantoea*, *Pseudomonas* and *Serratia*). In our study, in addition to the mentioned genera, *Stenotrophomonas*, *Delftia* and *Providencia* were also identified. The identification of more bacterial genera could be attributed to the use of the culturomic approach, which used the entire larva instead of dissected intestines. This avoids the loss of bacterial species during the process and allows the evaluation of the complete larval microbiota.

The IS **C**_**18**_**MImCl** modified the cultivable microbiota of the exposed larvae in relation to the non-appearance of certain species, when evaluated at different concentrations. For example, regarding to *Serratia marcescens*, *Enterobacter asburiae* and *Stenotrophomonas maltophila*, these were only found when larvae were exposed to LC_20._ In the case of **C**_**16**_**MImMeS**, this was found only for *Burkholdeira* sp. Independent of the applied IS concentration, **C**_**16**_**MImMeS** did not exert any influence on the cultivable microbiota. Changes in the microbiota of culicids have been related to the food supply, habitat and genetic factor, among others^19^.

Nowadays, the functional and physiological role of bacterial species in larvae is still not fully understood, once the studies about microbiota were mainly focused on adult mosquitoes. In this way, some species of *Serratia* and *Enterobacter* produce hemolytic enzymes, being related to the digestion of blood in adult females and probably acquired during the larval stage^20,21^. Herein, *Stenotrophomonas maltophila* was found in larvae of a laboratory *Ae*. *aegypti*. This bacterial species was found in great abundance in the gut of adult *Ae*. *aegypti* caught in the field^22^, but its presence in larvae has not yet reported.

When verifying the action of IS **C**_**18**_**MImCl** through the cultivable microbiota, RISA demonstrated a Shannon’s diversity (H′) lower than that of the control group for both LC_99_ and LC_50_. For **C**_**16**_**MImMeS**, the LC_99_ resulted in a lower H′ and the LC_50_ presented a similar result as the control group.

The phylogenetic trees (Figs 2 and 3) suggested that there were changes in the bacterial community, since some species were eliminated and others emerged (Operational Taxonomic Unities does not always represent the same species)^23^. A total change in the microbiota might occur, which could promote larval death or increase the susceptibility towards IS which was confirmed by changes in the bacterial communities inside the larvae after exposure to IS.

As these alterations in the microbiota are not necessarily a determining factor in the larval death, the effect of IS on larvae without their microbiota was studied. After 48 h, significant differences were observed when axenic larvae were exposed to the IS, when compared with the group of non-axenic larvae. This suggested that bacterial communities interact with the IS, which potentializes their larvicidal action. The antibacterial IS could kill or reduce the proliferation of part of the microbiota and favor the multiplication of pathogenic bacteria. This hypothesis was further corroborated by the survival of axenic larvae after IS exposure, which became susceptible after being re-colonized by bacteria. In this way, one can speculate that the IS interact preferentially with bacteria acquired from the environment than with those that are transmitted vertically. Within this context, it is worth remembering that these results were obtained with larvae reared in laboratory. Therefore, this dynamic could change for populations of larvae from the field, once these mosquitoes have a more diversified microbiota than those from the laboratory^24^.

A benchmark larvicide was also tested for comparison, since it may be that all larvicides have some kind of interaction with the microbiota, thus triggering the death of the larvae. Temephos was chosen for this purpose, which showed high mortality in both non-axenic and axenic larvae. The mode of action of this molecule is well known, acting in the central nervous system of the larvae and inhibiting cholinesterase^25^. As far as we know, there is no study that relates chemical insecticides and mosquito microbiota. In the case of biological insecticides, larvae change their microbiota when intoxicated with the entomopathogenic bacterium *Bacillus thuringiensis israelensis*^26^. This study demonstrated that *Bti*-tolerant larvae (with death above 12 h of post-exposure) have low diversity and large inter-individual differences. It was also described that the gut bacteria of larvae aid in the degradation of toxins produced by *Bti*, therefore decreasing their effectiveness^27^. The fungus *Beauveria bassiana* also interacts with the *Anopheles stephensi* microbiota, accelerating mosquito death. In axenic mosquitoes, the death slowed down, as seen in our study^28^.

Thus, when larvae are treated with IS, the microbiota of mosquitoes can play an important role in their control. This report is the first study using MALDI-TOF mass spectrometry and culturomics for the identification of cultivable microbiota of larvae in *Ae*. *aegypti*. It is also the first report related to a potential chemical larvicide for the control of *Ae*. *aegypti* that can be potentialized by the microbiota, providing a first insight into the IS-microbiota interaction.

## Material and Methods

### Maintenance of mosquitoes

Larvae of the *Ae*. *aegypti* Rockefeller strain were used for all bioassays. They were reared at 28 °C, a 12-hour light/dark photoperiod, and an 80% relative humidity (RH). Cat cub food was offered twice a week for larvae development. After the development of pupae, they were daily removed and placed in cages for adult emergence. Adult males were fed on honey solution (10%), while females were fed on sheep’s blood (Newprov®) through a membrane for eggs development.

### Imidazolium salts

Nuclear magnetic resonance (NMR) spectra were recorded on a Bruker (400 MHz) equipment at ambient temperature. The chemical shifts are given in parts per million (ppm) and referenced to the residual solvent signal (CDCl_3_ = 7.26 (^1^H), 77.16 (^13^C)). Attenuated total reflection Fourier transform infrared spectroscopy (ATR-FTIR), in the mid infrared range (4000–500 cm^−1^), was performed on an ALPHA-P compact Bruker FTIR spectrometer. High-resolution mass spectrometry spectra were recorded on an electrospray ionization (ESI) Q-Tof Micro^TM^ equipment (Micromass, Manchester, UK) in the positive mode.

1-Methyl-3-*n*-octadecylimidazolium chloride (**C**_**18**_**MImCl**) was purchased at CJC CHINA JIE CHEMICAL. *n*-Hexadecyl methanesulfonate was synthesized according to previously reported procedures; and the spectral data were in agreement with those reported^29^. A modified literature procedure was used for the synthesis of 1-*n*-hexadecyl-3-methylimidazolium methanesulfonate (**C**_**16**_**MImMeS**)^30^. The mixture of 1-hexadecyl methanesulfonate (11.9 mmol, 1.00 equiv.) and 1-methylimidazole (11.9 mmol, 1.00 equiv.) was stirred and heated at 100 °C for 2 h. Next, the crude product was recrystallized from hot ethyl acetate.

**C**_**16**_**MImMeS** was obtained in 89% yield as a white solid. ^1^H NMR (400 MHz, CDCl_3_) δ 9.74 (s, 1H), 7.45 (t, *J* = 1.6 Hz, 1H), 7.30 (t, *J* = 1.7 Hz, 1H), 4.20 (t, *J* = 7.4 Hz, 2H), 4.00 (s, 3H), 2.72 (s, 3H), 1.83 (m, 2H), 1.26–1.20 (m, 26H), 0.83 (t, *J* = 6.8 Hz, 3H). ^13^C NMR (101 MHz, CDCl_3_) δ 137.98, 123.61, 121.76, 49.95, 39.65, 36.38, 31.88, 30.23, 29.89–29.22 (9C), 28.98, 26.23, 22.65, 14.09. ESI(+)-MS: C_20_H_39_N_2_^+^ - calculated: 307.3108, obtained: 307.4160 (See Supplementary Information).

Before use, the IS were dried under vacuum for 5 h at 60 °C to remove residual water.

### Larvae exposition to IS

Based on the report of Goellner *et al*.^7^, the IS LC_99_ (5 µg/mL), LC_50_ (2.5 µg/mL) and LC_20_ (1.25 µg/mL) were tested to study the influence of IS on the larvae microbiota of *Ae*. *aegypti*. For the evaluation of cultivable microbiota; four groups of larvae were used for each IS: control (non-exposed), LC_20_, LC_50_ and LC_99_. For the diversity index experiments, the LC_99_ and LC_50_ were used. Eggs of *Ae*. *aegypti* were placed to hatch in 1 liter of distilled water. After hatching and visualizing the larvae, they were kept in an incubator (Tecnal TE-401) at 28 °C and 80% RH, and fed with previously sterilized cat puppy food. After reaching the earlier L_2_ and later L_3_ stages, the larvae were removed and used as control group (non-exposed). At the same stages, the larvae were subjected to IS treatments, using 25 larvae for each group (LC_20_, LC_50_ and LC_99_). Each group of 25 larvae was disposed in a sterile glass container with 100 mL of autoclaved distilled water. Only larvae that showed non-lethargy after IS treatment were used in further studies.

### Isolating and identifying cultivable bacteria

This method comprehends a culturomic approach^31^, which was adapted for healthy adult mosquitos of *Ae*. *aegypti* (unpublished data) and then used in larvae. The experiments described below were carried out in triplicate, and each group consisted of five larvae (non-treated, LC_99,_ LC_50_ and LC_20_). The larvae were washed in 70% ethanol (3 minutes), and rinsed three-times with ultra-pure water to assure a high level of disinfection. The larvae were macerated in 2 mL of sterile distilled water, submitted to a pre-incubation medium composed of brain-heart infusion broth (10 g/L), yeast extract (5 g/L), bacteriological peptone (5 g/L) and 5% of sheep’s blood (Newprov®) in 1000 mL of distilled water, and incubated at 28 °C during a 30-day period. Aliquots (1 mL) were removed from the pre-incubation at the beginning of the experiment and after every five days. All aliquots were serially diluted until 1 × 10^−10^, and each dilution was sown in Petri dishes containing trypticase soy agar. From the growth of bacterial colonies, each colony morphotype was transferred to a new Petri dish and subjected to its identification.

Each colony was submitted, in duplicate, to matrix-assisted laser desorption/ionization time of flight mass spectrometry (MALDI-TOF MS). The samples were placed on the target plate (96 target MSP polished steel, Bruker Daltonics) and covered with the α-cyano-4-hydroxycinnamicacid matrix (Bruker Daltonics) diluted in 50% acetonitrile (v/v), 2.5% trifluoroacetic acid (v/v) and Milli-Q ultrapure water, followed by a drying period at room temperature. The calibration was performed with the bacterial test standard reagent (Bruker Daltonics). All spectra were recorded in the linear mode, within a range of 2,000 to 20,000 daltons. The peaks of each spectrum were compared to the database (containing 5,623 reference spectra) and the score considered ideal for this study was higher or equal to 2.0 (identification at the species level). When there was no identification of the species, the colonies were tested for at least three times. The spectra were generated using Biotyper Software (Bruker Daltonics, Germany).

### Cultivation-independent analysis

For the microbial diversity assessment of larvae exposed to IS, the ribosomal intergenic space analysis (RISA) technique was applied, using the protocol of Solaiman & Marschner^32^. Three groups of five larvae were used: control (non-exposed), LC_50_ and LC_99_. All tests were made in triplicate. Finally, the larvae were washed subsequently in 70% ethanol for 3 min and three times in sterile ultra-pure water for a superficial disinfection. Each larva was macerated in 2 mL of sterile distilled water and subjected to the PowerSoil® kit for total DNA extraction, according to the manufacturer’s guidelines, and quantified through NanoDrop®. Extracted DNA samples were submitted to PCR, using the oligonucleotide primers **23S** (5′ GGGTTBCCCATTCRG 3′) and **1406** (5′ TGYACACACCGCCCGT 3′). These primers amplify a gene rRNA operon region between small (16S) and large (23S) sub unities, termed intergenic spacer region. Each sample was prepared with 5 µL of the 5X tampon, 0.2 mM of dNTP, 10 µM of primers, Taq DNA polymerase 1U, 50 ng extracted DNA and Milli-Q sterile water to complete the volume of 50 µL. The process initiated with an initial denaturation at 94 °C for 2 min, followed by 35 denaturation cycles at 94 °C for 30 sec, followed by annealing at 51 °C for 30 sec, extension at 72 °C for 1 min, and at last finalextension at 72 °C for 5 min. The PCR products (30 µL of each PCR reaction) were placed on a 3% agarose gel in TBE 0.5X, and stained with GelRed® containing bromophenol. For the electrophoresis, 75 V and 120 mA were applied for 3 h.

### Production of axenic larvae

**Figure 7:**
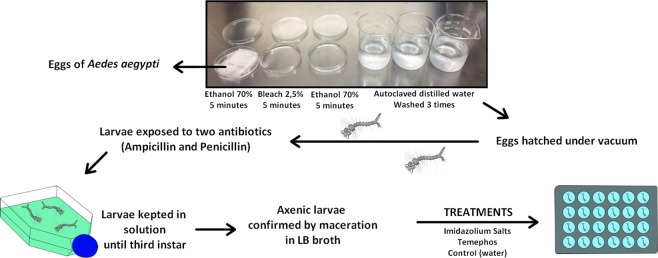
Experimental design for the production of axenic larvae of *Aedes aegypti*.

The protocol of Correa *et al*. was modified for the production of axenic larvae (Fig. 7)^13^. Thus, a filter paper strip with eggs of *Ae*. *aegypti* was disinfected by immersion for 5 min in 70% ethanol, followed by 5 min in aqueous 2.5% sodium hypochlorite and again in 70% ethanol for 5 min. Finally, the eggs were washed three times with autoclaved distilled water. For hatching, the eggs were immersed in 250 mL of phosphate-buffered saline solution (PBS) and exposed to vacuum at 25 Hz for 15 min at room temperature. After hatching, the larvae were exposed to two different antibiotics, supplementing the solution of hatching eggs with a final concentration of 75 µg/L of ampicillin and 125 µg/L of penicillin, following an incubation period of 4 h at 28 °C. Next, the larvae were transferred to a new medium containing 500 mL of distilled water and 4 g of *E*. *coli* agar, and then stored at a temperature of 28 °C, until reaching the third instar. For axenia control, 15 larvae were separated at the beginning of the treatment (first instar) and another 15 larvae at the end of the treatment (third instar), obtaining two control groups of triplicates of five larvae. They were then muddled, submitted to 14 mL of LB broth, and left for 48 h at 28 °C. For comparison purposes, the same was done with the laboratory larvae and the axenia was verified by bacterial growth in the broth by the turbidity of the medium.

### Axenic larvae susceptibility

Two groups of L3 axenic larvae were exposed to an IS LC_99_ concentration. Another group was exposed to Temephos to check if the absence of microorganisms in the larvae affected the efficacy of this larvicide. These bioassays were conducted in 24-well plates, in triplicate and repeated four times. For each well, 10 larvae were laid in 2 mL of the solutions (LC_99_ = 5 µg/mL for **C**_**18**_**MImCl** and **C**_**16**_**MImMeS**; and LC_99_ = 6 µg/L for Temephos), using also standard lab larvae as a control group. The larval mortality was determined after 24 and 48 h.

### Susceptibility assays with re-colonized axenic larvae

**Figure 8:**
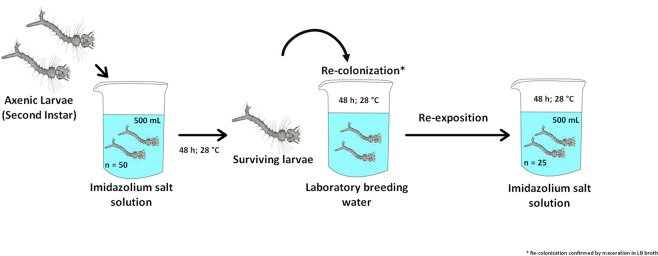
Experimental design for the treatment of axenic larvae of *Aedes aegypti* with IS, their re-colonization with bacteria, and subsequent second treatment with IS.

Axenic larvae that were exposed to the IS treatments and remained alive were exposed to usual larvae breeding water (Fig. 8). Subsequently, they were exposed again to the IS to check if the reintroduction of bacteria could influence in their mortality. The same procedures described above were followed for the production of axenic larvae. For both IS, fifty second-instar (to ensure a number of live larvae until the end of the experiment) larvae were exposed to 500 mL of an IS LC_99_ solution for 48 h at 28 °C (mortality was observed after 24 and 48 h). Afterwards, all the non-lethargic surviving larvae were laid into a breeding place with 500 mL of water, and incubated for 48 h at 28 °C (48 h–96 h). The number of re-exposed larvae was standardized to 25 before the second exposition to an IS solution, and the mortality was observed after 120 and 144 h. Axenia and re-colonization was confirmed by exposing 5 larvae to 14 mL of LB broth for 48 h at 28 °C. This test was performed when the larvae reached the second instar, before they were exposed for the first time to the IS, and after being fed for 48 h with the standard laboratory larvae breeding medium. All procedures were made in triplicate (50 axenic larvae; 25 recolonized per replicate).

### Statistical analysis

For the culture-independent assay, it was possible to verify the similarity between fingerprints generated by RISA. The bands were normalized creating a pattern of absence-presence of bands and generating a binary matrix, which was used to calculate the Dice coefficient. The results were grouped by the unweighted pair group method with arithmetic mean (UPGMA).

The diversity of bacteria communities was calculated through the diversity Shannon’s index (H′). For this, the intensity of the fingerprint bands was determined by densitometric analysis. The equation used was: H′ = −**∑** (***n***_***i***_/***N***) **log** (***n***_***i***_/***N***), where *n*_*i*_ is the amount related to the peak intensity of each band and N is the sum of all peak intensities, obtained through the densitometric curve^33^.

For the axenic larvae treatments and Shannon Index (H′), significant effects of the IS on larvae mortality were determined by two-way variance analysis (ANOVA). The differences between the groups were determined using Tukey’s test, considering the significance level P < 0.05.

## Supplementary information

Supplementary information

## Data Availability

No datasets were generated or analysed during the current study.
