# Supplementary material for: Microbiota potentialized larvicidal action of imidazolium salts against Aedes aegypti (Diptera: Culicidae)
Source: Sci Rep. 2019 Nov 7;9:16164. doi: 10.1038/s41598-019-52687-4 (PMC6838459; doi:10.1038/s41598-019-52687-4)
Supplement: Supplementary file 1 — Supplementary information [file 41598_2019_52687_MOESM1_ESM.docx]

# Supplementary Information

**Microbiota potentialized larvicidal action of imidazolium salts against *Aedes aegypti* (Diptera: Culicidae)**

Harry Luiz Pilz-Junior1, Alessandra Bittencourt de Lemos1, Kauana Nunes de Almeida2, Gertrudes Corção1, Henri Stephan Schrekker2,*, Carlos Eugenio Silva1, Onilda Santos da Silva1,*

1Department of Microbiology, Immunology and Parasitology, Institute of Basic Health Sciences, Universidade Federal do Rio Grande do Sul, Rua Sarmento Leite 500, Porto Alegre, RS, 90050-170, Brazil.

2Laboratory of Technological Processes and Catalysis, Institute of Chemistry, Universidade Federal do Rio Grande do Sul, Av. Bento Gonçalves 9500, Porto Alegre, RS, 91501-970, Brazil.

*Corresponding authors: [onilda.silva@ufrgs.br](mailto:onilda.silva@ufrgs.br) (O.S.S.) [henri.schrekker@ufrgs.br](mailto:henri.schrekker@ufrgs.br) (H.S.S.);

.


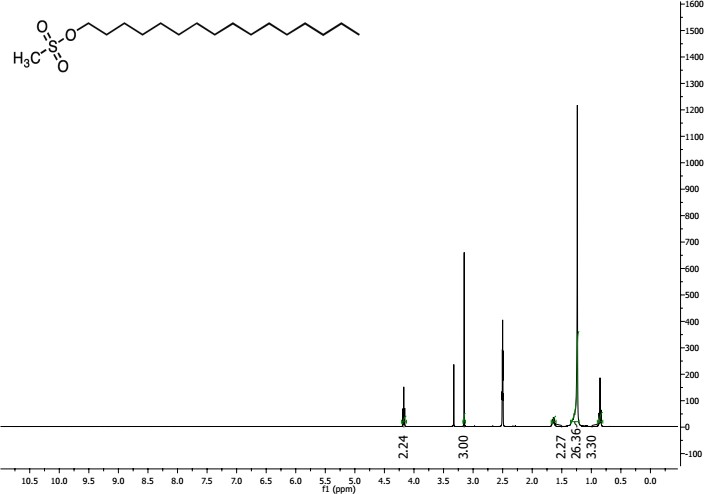


**Figure S1.** 1H NMR spectrum of *n*-hexadecyl methanesulfonate (400 MHz, DMSO-d6).


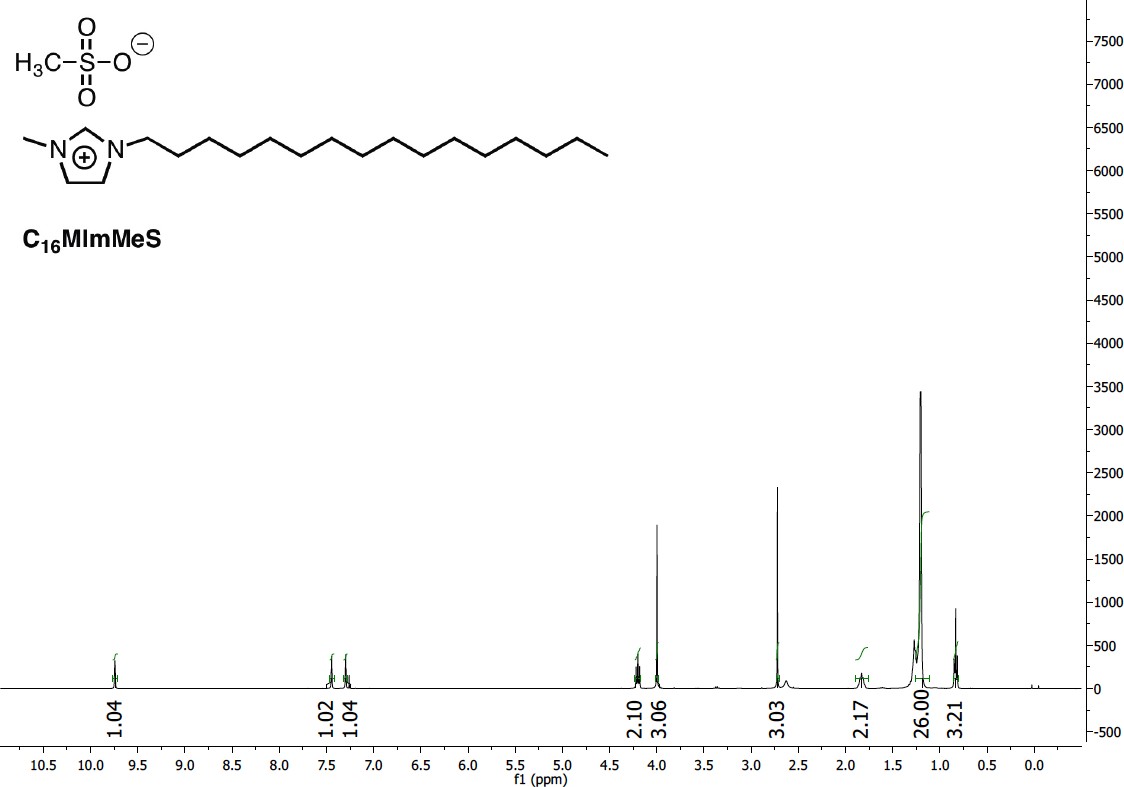


**Figure S2.** ^1^H NMR spectrum of **C16ImMeS** (400 MHz, CDCl3).


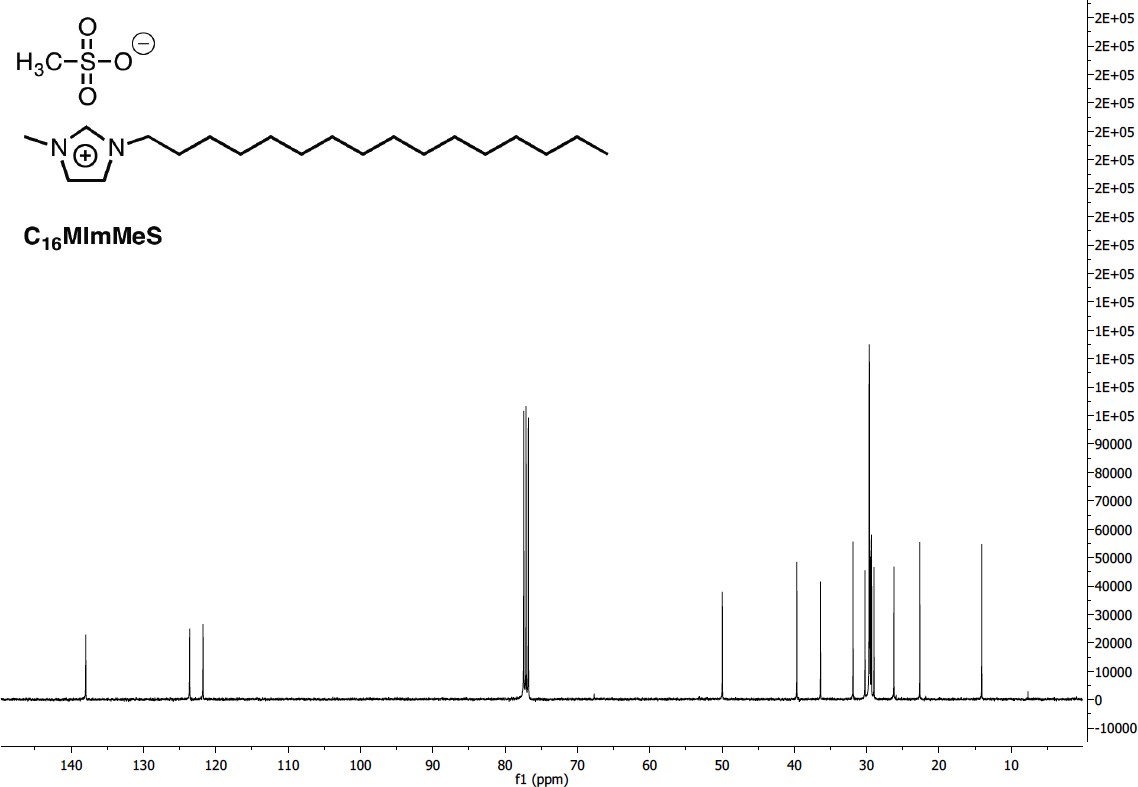


**Figure S3.** ^13^C NMR spectrum of **C16ImMeS** (101 MHz, CDCl3).


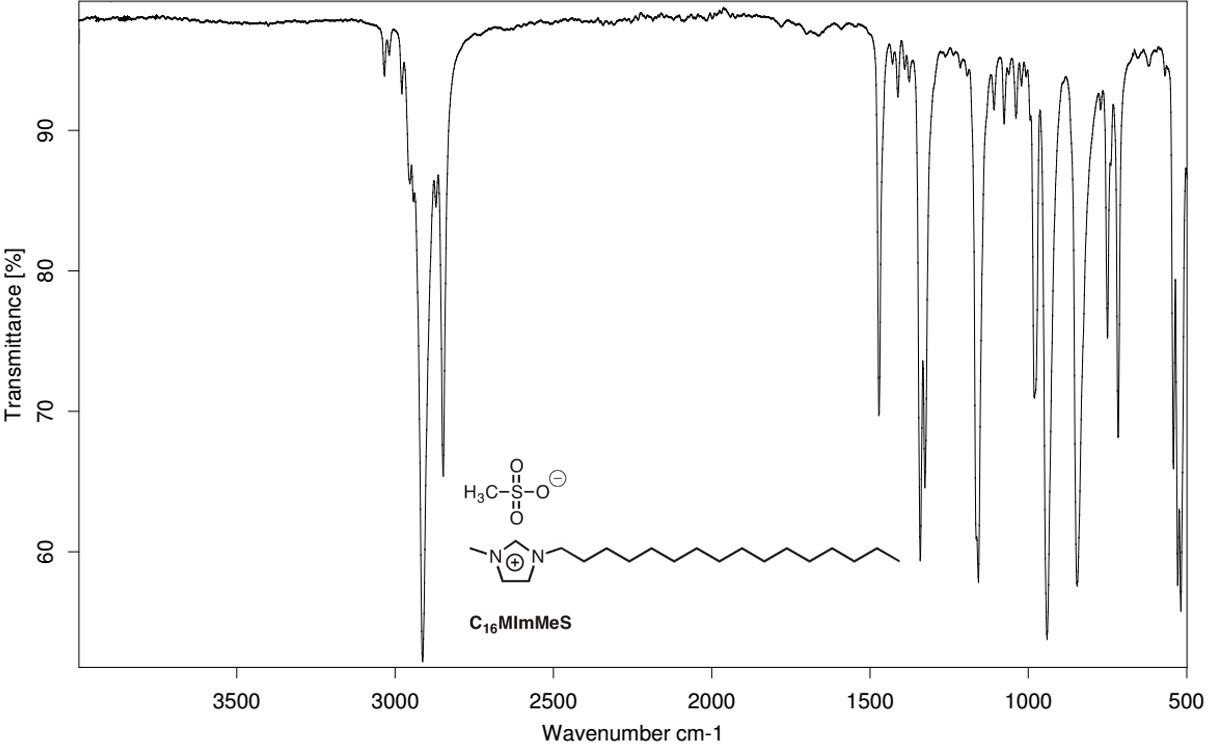


**Figure S4.** FTIR spectrum of **C16ImMeS**.


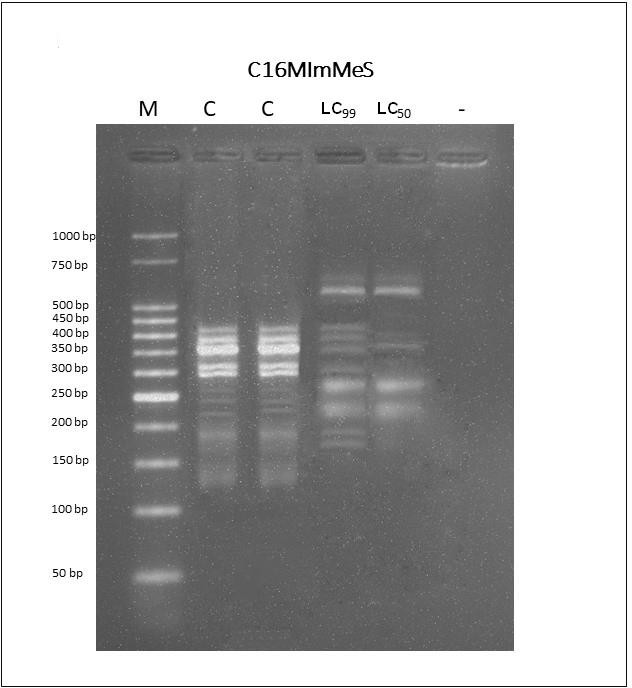


**Figure S5**. Full-length gel from the treatments with the imidazolium salt **C16MImMeS.** M = Molecular Ladder; C = Control; LC99 = Lethal Concentration 99%; LC50 = Lethal Concentration 50%; - = negative control.


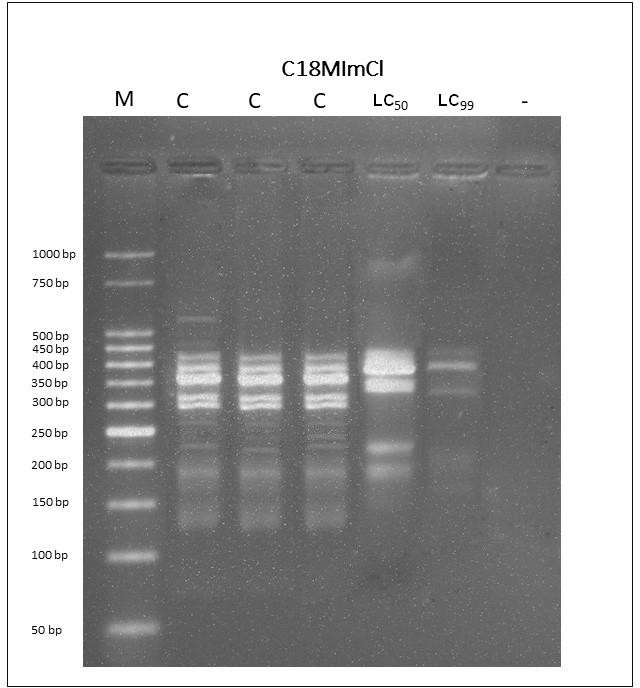


**Figure S6**. Full-length gel from the treatments with the imidazolium salt **C18MImCl.** M = Molecular Ladder; C = Control; LC99 = Lethal Concentration 99%; LC50 = Lethal Concentration 50%; - = negative control.
